# Large Cell Neuroendocrine Carcinoma of the Cervix With Extensive Metastases

**DOI:** 10.1002/kjm2.70100

**Published:** 2025-09-01

**Authors:** Di‐Ping Yu, Li‐Mei Sun

**Affiliations:** ^1^ Department of Pathology The Puer People's Hospital Puer Yunnan Province China; ^2^ Department of Respiratory and Critical Care Medicine The Puer People's Hospital Puer Yunnan Province China

1

Large cell neuroendocrine carcinoma (LCNEC) of the cervix is a rare pathological subtype in cervical cancer, which has a worse prognosis, accounting for less than 0.2% of all cervical cancers [1]. The development of this tumor is strongly associated with persistent infection with high‐risk human papilloma virus (HPV) types, especially HPV‐16 and HPV‐18 [2]. Given its rarity and the absence of randomized trials, the diagnostic and therapeutic management of this tumor is complicated. Here we report a case of cervical LCNEC with extensive metastases.

A 45‐year‐old female patient presented with vaginal bleeding accompanied by lower abdominal pain for 1 month. Her menstrual cycle was regular, with a history of two pregnancies and childbirth. She denied any family history of genetic disorders or cancer. HPV typing by polymerase chain reaction was positive for HPV‐18. Physical examination revealed a cauliflower‐like cervical mass approximately 4 cm in diameter. Abdominal ultrasound (US) showed a large heterogeneous mass of 11.0 × 6.9 cm on the left ovary and 14.6 × 7.0 cm on the right ovary (Figure 1A). Abdominal magnetic resonance imaging (MRI) revealed two solid masses in the liver (Figure 1B). Single Photon Emission Computed Tomography/Computed Tomography (SPECT/CT) showed multiple lesions with increased bone metabolism (Figure 1C), and bone metastasis was considered. Subsequently, a cervical biopsy was carried out. The histologic features displayed organoid, solid‐sheet, or trabecular growth patterns. Large neoplastic cells displayed moderate to abundant cytoplasm, vesicular nuclei, and conspicuous nuclei, with a high mitotic rate (Figure 1D,E). Immunohistochemical stains exhibited positive reactivity for Pan‐cytokeratin, synaptophysin (Figure 1F), CD56, INSM1 (Figure 1G), p16 (Figure 1H), and weakly for CgA. Immunostaining for CK5/6, CK20, WT‐1, PAX8, p40, and p63 were negative. The Ki‐67 proliferative index was 80% (Figure 1I). Based on these histologic and immunohistochemical findings, the diagnosis of LCNEC was rendered. The patient received adjuvant chemotherapy with 6 cycles of etoposide and cisplatin. Due to a worsening of the patient's condition, the patient died 14 months after the initial diagnosis.

**FIGURE 1:**
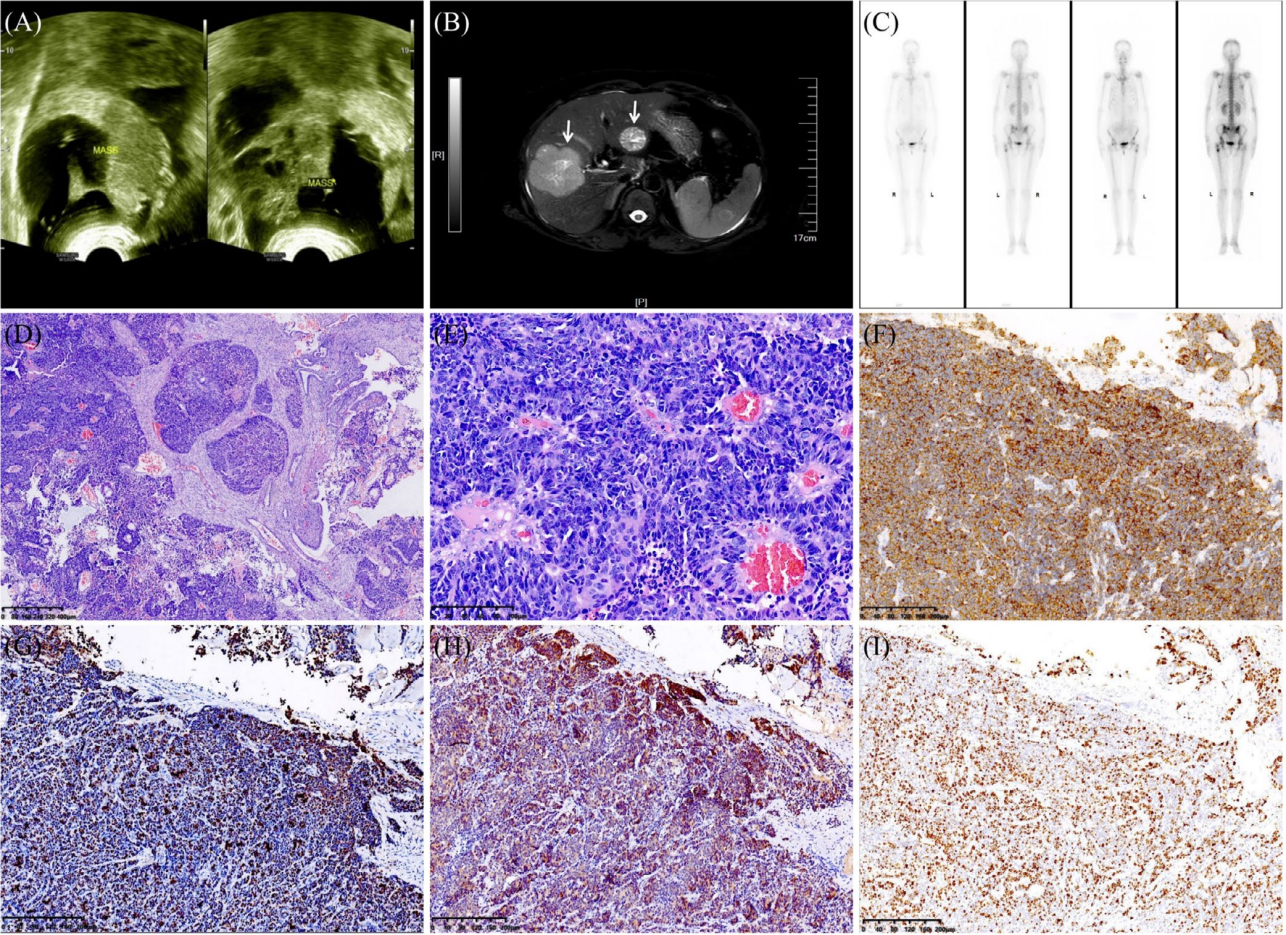
(A) Abdominal ultrasound (US) showed a large heterogeneous mass of 11.0 × 6.9 cm on the left ovary and 14.6 × 7.0 cm on the right ovary. (B) Abdominal magnetic resonance imaging (MRI) revealed two solid masses in the liver (arrow). (C) Anterior and posterior whole body ^99^mTc MDP bone scan planar SPECT/CT images revealed abnormal increases in the radioactivity of the left scapula, sacroiliac joint, ilium, and proximal femur. (D) At microscopic examination, this lesion displayed organoid, solid‐sheet, and trabecular growth patterns (H&E, original magnification × 40). (E) Large neoplastic cells displayed moderate to abundant cytoplasm, nuclear pleomorphism, vesicular nuclei, and conspicuous nuclei, with a high mitotic rate (H&E, original magnification × 200). (F–H) Immunohistochemical staining positive for synaptophysin, INSM1, and p16 (original magnification × 100). (I) The Ki‐67 proliferative index was 80%.

Cervical LCNEC is a highly aggressive tumor with a median age of onset of around 36 years. Clinically, it manifests as abnormal uterine bleeding and leukorrhea. Compared with common types of cervical malignant tumors, its signs and clinical manifestations are not specific, but cervical LCNEC is highly invasive, and it is easy to metastasize early through blood or lymphatic routes. In addition, cervical LCNEC has neuroendocrine functions, but most hormones secreted are inert, with only a minority of patients exhibiting corresponding neuroendocrine symptoms (Cushing's syndrome, carcinoid syndrome, hypoglycemia, syndrome of inappropriate antidiuretic hormone secretion, hypercalcemia) [3]. The typical pathological features are the primary basis for diagnosing LCNEC. According to the WHO classification, LCNEC exhibits characteristic neuroendocrine morphology with large cell size and positive immunohistochemical staining for neuroendocrine markers such as synaptophysin, CgA, CD56, and INSM1 [4]. Considering the low degree of differentiation of LCNEC, the combined labeling of multiple markers is more meaningful for the diagnosis. Cervical LCNEC is frequently linked to the high‐risk HPV, which is also believed to play a crucial role in the progression of the tumor. Immunohistochemical staining indicated p16, with positive p16 staining indicating persistent infection by the high‐risk HPV16 and/or HPV18.

As a rare malignant tumor of the cervix, the treatment of cervical LCNEC lacks standardization, and currently, radical surgery combined with radiotherapy and chemotherapy is the most common treatment method. Regardless of the stage of the cervical tumor, systemic chemotherapy is recommended. Platinum‐based drugs have been proven to improve the patient's overall survival (OS) or progression‐free survival (PFS). The Society of Gynecologic Oncology (SGO) has recently formulated guidelines for women with cervical neuroendocrine carcinomas, suggesting a multimodal approach involving neoadjuvant chemotherapy based on etoposide and platinum (EP regimen) [5].

This case suggests that cervical LCNEC is a rare and highly aggressive malignancy with early metastasis. Although multimodal treatment plans are used for this disease, the prognosis still falls short of expectations. Therefore, early diagnosis and timely treatment after diagnosis are very important for patients.

## Conflicts of Interest

The authors declare no conflicts of interest.

## Data Availability

Data sharing is not applicable to this article as no new data were created or analyzed in this study.
